# Challenges and Prospects of Travel Medicine Practice in Nigeria: A Cross-Sectional Survey of Travel Medicine Practitioners

**DOI:** 10.7759/cureus.104901

**Published:** 2026-03-09

**Authors:** Babalola A Ibisola, Adegboyega O Alao, Bukola Adeniyi

**Affiliations:** 1 Family Medicine, Triverge Healthcare, Ibadan, NGA; 2 Family Medicine, Lagoon Hospitals, Lagos, NGA; 3 Family Medicine, Q-Life Family Clinic, Lagos, NGA

**Keywords:** challenges, international and travel medicine, nigeria, prospects, travel medicine, travel medicine practice

## Abstract

Background: Travel medicine is a relatively new area of practice in Nigeria and most African countries. Even though a huge number of Nigerians engage regularly in both international and in-country travels, there is a dearth of travel medicine practitioners in the country who can serve this population. This study was conducted to help in the identification of some of the challenges and prospects of travel medicine practice in Nigeria.

Methods: This was a cross-sectional study. Data collection was done via electronically distributed questionnaires to travel medicine practitioners in Nigeria. There were 52 respondents but total of 33 respondents were accepted based on the inclusion criteria and analyzed. The analysis was done using IBM SPSS Statistics software, version 26.0 (IBM Corp., Armonk, NY, USA).

Results: The median age of respondents was 42.5 years, and 18 (54.5%) were males, 27 (81.8%) practiced Christianity, and 19 (57.6%) had their practice in Lagos. Thirty (90.9%) were doctors, and 23 (69.7%) of them had no travel health certification. Highlighted challenges of travel medicine clinical services include vaccine supply (16, 48.5%), vaccine procurement (13, 42.4%), and vaccine storage (16, 42.5%). Twenty-one (63.6%) respondents highlighted poor patronage as a challenge facing the practice of travel medicine, while 28 (84.9%) agreed that the newness of travel medicine is also a challenge, and 27 (81.8%) agreed that travel medicine has not yet been widely recognized as a useful clinical service. Twenty-four (72.7%) and 30 (90.9%) suggested that the use of information technology and the use of Electronic Management of Immunization Data (EMID) to monitor vaccination, respectively, would improve the prospects of travel health practices in Nigeria. Twenty-six (78.8%) believed that there is a positive outlook for travel medicine in the post-COVID era.

Conclusion: This research highlights the necessity for targeted efforts towards addressing the issues faced by travel medicine practitioners in the country. The study identified the primary challenges to be the novelty of the specialty and the lack of recognition of its importance to travelers. As for potential solutions, it is suggested that extensive utilization of technology in various forms will enhance the practice of travel medicine in Nigeria.

## Introduction

Travel medicine is the field of medicine concerned with the promotion of health for the people, culture, and environment of regions being visited, in addition to the prevention of disease or other adverse health outcomes in the international traveler. It focuses primarily on pre-travel preventive care [1]. Travel medicine is a rapidly evolving, highly dynamic, multidisciplinary specialty that requires expertise on various travel-related illnesses, as well as up-to-date knowledge on the global epidemiology of infectious and non-infectious health risks, health regulations and immunization requirements in various countries, and the changing patterns of drug-resistant infections [2].

The growth of travel medicine in Africa has been slow, though it has been gaining some traction in Nigeria with the launch of the Nigerian Society of Travel Medicine (NSTM). The NSTM was launched in Lagos on 20^th^ February 2020. The vision of the society is to promote safe and healthy travel within and outside Nigeria for travelers while educating those traveling to areas with high circulation of infectious diseases [3]. As of 2022, when the survey was conducted, there were 50 financial members in the society, and the number continues to grow.

The field of travel medicine has evolved rapidly and is now recognized as a distinct specialty, meeting the needs of an ever-increasing number of travelers. Historically, the focus was on tourists from Europe and North America, while low- and middle-income countries, where infectious disease risks are higher, were neglected, although this paradigm is changing [4-6].

Although travel medicine experienced a significant decline during the COVID-19 pandemic, previous global pandemics indicate that demand for pre-travel care will return. Increased awareness of hand hygiene and respiratory etiquette may encourage travelers to seek professional health advice more frequently in the future [7, 8].

Despite its importance, travel medicine has limitations. One global challenge is providing travel-related healthcare to persons with disabilities, a population largely overlooked in travel medicine literature [9]. According to the World Health Organization, one in seven people globally lives with a disability, many of whom travel [10]. Even literature addressing disabled sporting events often fails to provide disability-specific travel guidance [6,11].

These challenges vary across regions and countries, forming the rationale for this study. Hence, the objective of this study was to evaluate the challenges and prospects of travel medicine practice in Nigeria.

## Materials and methods

This study is a descriptive cross-sectional study, which employed an electronic method of data collection. In 2022, a Google questionnaire (Google Inc., Mountain View, CA, USA) online link was developed and shared electronically on social media, especially WhatsApp (Meta Platforms, Inc., Menlo Park, CA, USA), to all healthcare practitioners of the NSTM. This was a total sampling of all 85 financial and non-financial members of NSTM. This was done after ethical approval was sought and obtained from the National Health Research Ethics Committee (NHREC) of Nigeria. The ethical approval number is NHREC/01/01/2007-21/02/2022. Implied consent was obtained by filling out the online anonymous questionnaire.

The survey tool, an online questionnaire, contained various questions grouped into six distinct sections to collect data such as the demographic characteristics, professional information, challenges in setting up a travel health clinic, challenges of travel medicine practice in Nigeria, the services provided at a travel medicine clinic, and the prospects of travel medicine practice in Nigeria (Appendix A). The survey tool was developed by the authors, peer-reviewed, and face-validated by senior colleagues before use.

The responses were collated and sorted subsequently. There were 52 respondents (response rate was 61%), but responses from healthcare practitioners who do not practice travel medicine or have not been in active practice of travel medicine in the preceding six months, or those who practiced travel medicine outside Nigeria, were excluded and not analyzed.

A total of 33 respondents were accepted based on the inclusion criteria and analyzed. The data analysis was done using IBM SPSS Statistics software, version 26.0 (IBM Corp., Armonk, NY, USA). The results of numerical variables like age were described as the median. Categorical variables were represented as numbers and percentages for descriptive analysis.

## Results

The analysis was based on responses obtained from 33 participants included in the study.

Socio-Demographic Characteristics

As shown in Table 1, the median age of respondents was 42.5 years, with 16 (48.5%) aged between 41 and 60 years. Eighteen (54.5%) of the respondents were male. Twenty-seven (81.8%) identified as Christians, and 25 (75.7%) were of Yoruba ethnicity. Nineteen (57.6%) practiced in Lagos State, and 17 (51.5%) held a postgraduate fellowship qualification.

**Table 1:** Socio-demographic characteristics of the respondents

Variables	Frequency	Percentage (%)
Age		
≤ 40 years	12	36.4
41 - 60 years	16	48.5
> 60 years	4	12.1
No response	1	3.1
Median (IQR)	42.50 (39.00 – 55.50) years	
Range	36.00 – 70.00 years	
Gender		
Male	18	54.5
Female	15	45.5
Religion		
Christianity	27	81.8
Islam	6	18.2
Ethnicity		
Yoruba	25	75.7
Igbo	2	6.1
Others	6	18.2
Place of Practice		
Lagos	19	57.6
Oyo	6	18.2
Ogun	2	6.1
Ondo	1	3
Enugu	2	6.1
Plateau	2	6.1
Kaduna	1	3
Educational Qualification		
Fellowship (West African, National, Royal, etc.)	17	51.5
First degree	5	15.2
Masters	9	27.3
Master's in view	1	3
Member of the West African College of Physicians (MWACP)	1	3

Professional Characteristics

Table 2 summarizes the professional profile of the respondents. Thirty (90.9%) were medical doctors, with 16 (48.5%) in family medicine being the most common specialty. The median duration of travel medicine practice was seven years, and 22 (66.7) had 10 years or less experience in the field. Notably, 23 (69.7%) did not possess any formal certification in travel health. Eight respondents (24.2%) reported owning a dedicated travel medicine clinic, while 14 (42.4%) indicated that they provided travel health services within a government hospital setting.

**Table 2:** Professional information of the respondents

Statement	Professional Information	Frequency	Percentage (%)
Profession	Medical doctor	30	90.9
	Nurse	1	3
	Pharmacist	2	6.1
Primary field of qualification	Clinical/Community pharmacist	1	3
	Family medicine	16	48.5
	Family/General practice	2	6.1
	General practice	7	21.2
	General practice & Women’s health	1	3
	General practice, Aviation medicine	1	3
	Hospital pharmacist	1	3
	Occupational health	1	3
	Public health and preventive medicine	1	3
	Public health physician	1	3
	Travel medicine	1	3
Duration of practicing travel medicine	≤ 10 years	22	66.7
	11 - 20 years	7	21.2
	> 20 years	4	12.1
	Median (IQR)	7.00 (4.00 – 14.50) years	
Type of travel medicine certification(s) possessed	ABC of Travel Health	1	3
	Authorized aviation medical examiner	1	3
	Certificate of attendance and participation (Liverpool School of Tropical Medicine (LSTM))	1	3
	Certificate in travel health	1	3
	Certificate of attendance	1	3
	Certificate training	1	3
	Certificate in Travel Medicine (CTM)	1	3
	Diploma in Travel Medicine and Certificate of Travel Health, International Society of Travel Medicine (CTH ISTM)	1	3
	South African Society of Travel Medicine (SASTM)	1	3
	Travel medicine certification, London School of Hygiene and Tropical Medicine (LSHTM)	1	3
	None	23	69.7
Having own travel medicine outfit (clinic)	Yes	8	24.2
	No	25	75.8
Category of travel medicine practice	Corporate	1	3
	General polyclinic	1	3
	Government clinic	1	3
	Group travel health practice	1	3
	Multinational	1	3
	Occasionally	1	3
	Private general practice & travel health practice	1	3
	Runs a travel health clinic at a government hospital	14	42.4
	Own travel health practice	6	18.2
	Work for a travel medicine clinic	1	3
	None	5	15.3

Challenges in Establishing Travel Medicine Services

As presented in Table 3, relatively few respondents identified structural or certification-related barriers as major challenges. Seven (21.3%) respondents agreed that locating an appropriate clinic site was challenging, while six (18.2%) identified start-up capital as a constraint. Five (15.1%) respondents agreed that the need for a physical structure posed a challenge. In contrast, 11 (33.3%) respondents agreed that the requirement to become a member of a travel medicine society was challenging. Additionally, nine (27.2%) respondents reported that the cost of certification examinations was a challenge, while 10 (30.3%) agreed that passing the licensing examination posed a difficulty. Overall, most respondents disagreed that these factors constituted significant barriers to the practice of travel medicine in Nigeria.

**Table 3:** Respondents’ responses on challenges of establishing travel medicine services

Statement	Responses	Frequency	Percentage (%)
Locating an appropriate clinic site	Strongly disagree	9	27.3
	Disagree	8	24.2
	Neutral	9	27.3
	Agree	5	15.1
	Strongly agree	2	6.1
Capital to start up the clinic	Strongly disagree	8	24.2
	Disagree	12	36.4
	Neutral	7	21.2
	Agree	4	12.1
	Strongly agree	2	6.1
Need for physical structure	Strongly disagree	4	12.1
	Disagree	14	42.4
	Neutral	10	30.3
	Agree	4	12.1
	Strongly agree	1	3.1
Need to become a member of the travel medicine society in my country	Strongly disagree	8	24.2
	Disagree	9	27.3
	Neutral	5	15.2
	Agree	8	24.2
	Strongly agree	3	9.1
Cost of writing the travel medicine certification examination	Strongly disagree	8	24.2
	Disagree	9	27.3
	Neutral	7	21.2
	Agree	8	24.2
	Strongly agree	1	3
Passing the travel medicine qualification (licensing) examination	Strongly disagree	8	24.2
	Disagree	8	24.2
	Neutral	7	21.2
	Agree	8	24.2
	Strongly agree	2	6.1

Challenges in the Practice of Travel Medicine

Table 4 highlights respondents’ perceptions of challenges related to active practice. A large majority, 28 (84.9%), agreed that the novelty of travel medicine within their communities was a major challenge. Poor patronage by travelers was also commonly reported, with 21 (63.6%) agreeing that it discouraged full engagement in practice. Similarly, 27 (81.8%) agreed that travel medicine had not yet been widely recognized as a useful clinical service. Practicing travel medicine as a stand-alone specialty was identified as a challenge by 24 (72.8%) of respondents. Conversely, most respondents (20, 60.6%) disagreed that providing repeated travel health advice was burdensome, and 25 (75.7%) disagreed that their city or place of practice was not yet ready for a travel medicine clinic.

**Table 4:** Respondents’ responses on challenges with the practice of travel medicine

Statement	Responses	Frequency	Percentage (%)
Travel medicine is still new/green to most people in their community of practice.	Strongly disagree	3	9.1
	Disagree	0	0
	Neutral	2	6.1
	Agree	16	48.5
	Strongly agree	12	36.4
Providing repeated travel advice to travellers is challenging/exhausting.	Strongly disagree	7	21.2
	Disagree	13	39.4
	Neutral	7	21.2
	Agree	6	18.2
	Strongly agree	0	0
The patronage of travellers is poor and discouraging to fully engage in travel medicine practice.	Strongly disagree	1	3
	Disagree	4	12.1
	Neutral	7	21.2
	Agree	14	42.4
	Strongly agree	7	21.2
Recognition of travel medicine as a useful clinic	Strongly disagree	2	6.1
	Disagree	2	6.1
	Neutral	2	6.1
	Agree	18	54.5
	Strongly agree	9	27.3
Practicing travel medicine alone without general medicine	Strongly disagree	1	3
	Disagree	3	9.1
	Neutral	5	15.2
	Agree	15	45.5
	Strongly agree	9	27.3
Readiness of the respondent’s city/place for a travel medicine clinic yet	Strongly disagree	11	33.3
	Disagree	14	42.4
	Neutral	5	15.2
	Agree	3	9.1
	Strongly agree	0	0

Travel Medicine Clinical Services

Table 5 presents responses related to clinical service delivery. Fourteen (42.5%) respondents agreed that their clinics could adequately meet the needs of all clients. Vaccine supply was identified as a major concern, with 16 (48.5%) strongly agreeing that it posed a serious challenge. Nearly half of the respondents (16, 48.5%) disagreed that cost was a barrier to potential travelers, and 14 (42.4%) disagreed that vaccine procurement was never an issue. Furthermore, 14 (42.4%) disagreed that their clinics could adequately cater to clients with disabilities, and 16 (48.5%) disagreed that vaccine storage was not a problem.

**Table 5:** Respondents’ responses on travel medicine clinical services

Statement	Responses	Frequency	Percentage (%)
Cost is an issue for potential travellers.	Strongly disagree	7	21.2
	Disagree	9	27.3
	Neutral	7	21.2
	Agree	6	18.2
	Strongly agree	4	12.1
Can our travel medicine clinics adequately meet the needs of every client?	Strongly disagree	9	27.3
	Disagree	3	9.1
	Neutral	7	21.2
	Agree	12	36.4
	Strongly agree	2	6.1
Are we able to cater to the disabled clients adequately?	Strongly disagree	7	21.2
	Disagree	7	21.2
	Neutral	7	21.2
	Agree	6	18.2
	Strongly agree	6	18.2
Vaccine procurement is never an issue.	Strongly disagree	5	15.1
	Disagree	9	27.3
	Neutral	6	18.2
	Agree	8	24.2
	Strongly agree	5	15.1
Vaccine supply is a serious issue.	Strongly disagree	4	12.1
	Disagree	9	27.3
	Neutral	4	12.1
	Agree	5	15.1
	Strongly agree	11	33.3
Vaccine storage is never an issue.	Strongly disagree	9	27.3
	Disagree	7	21.2
	Neutral	2	6.1
	Agree	8	24.2
	Strongly agree	7	21.2

Prospects of Travel Medicine Practice

As shown in Table 6, respondents were largely optimistic about the future of travel medicine in Nigeria. Most participants (29, 87.9%) agreed that the use of information technology would promote the growth of travel health services, while 24 (72.7%) agreed that information technology could significantly reduce the burden of initial clinic startup. Thirty (90.9%) respondents agreed that an electronic management of immunization data system could facilitate monitoring of travelers' vaccinations, similar to its application during the COVID-19 vaccination program. Additionally, 29 (87.9%) agreed that the development of software applications would enhance rather than hinder practice, and 26 (78.8%) believed that the post-COVID era would have a positive impact on travel health. Despite this optimism, more than half of respondents (20, 60.6%) reported that they did not consider themselves successful as travel health practitioners, and 28 (84.8%) indicated that travel medicine practice alone was insufficient to meet their economic needs.

**Table 6:** Respondents’ responses on prospects of travel medicine practice in Nigeria

Statement	Responses	Frequency	Percentage (%)
The use of information technology will help the growth of travel health in Nigeria.	Strongly disagree	1	3
	Disagree	1	3
	Neutral	2	6.1
	Agree	5	15.2
	Strongly agree	24	72.7
With the aid of information technology, the burden of initial start-up of a travel medicine clinic will significantly reduce.	Strongly disagree	0	0
	Disagree	1	3
	Neutral	8	24.2
	Agree	14	42.4
	Strongly agree	10	30.3
As was done with COVID-19 vaccination, Electronic Management of Immunization Data (EMID) can also help with travellers’ vaccination monitoring and thus make the tracking easy for a travel medicine clinic.	Strongly disagree	1	3
	Disagree	0	0
	Neutral	2	6.1
	Agree	17	51.5
	Strongly agree	13	39.4
The development of software applications for travel medicine practitioners will help to enhance their work rather than take away from it.	Strongly disagree	1	3
	Disagree	0	0
	Neutral	3	9.1
	Agree	14	42.4
	Strongly agree	15	45.5
Being successful as a travel health practitioner	Yes	13	39.4
	No	20	60.6
Travel medicine practice alone is sufficient to meet my economic needs.	Yes	5	15.2
	No	28	84.8
The post-COVID era will have a positive impact on travel health.	Strongly disagree	0	0
	Disagree	0	0
	Neutral	7	21.2
	Agree	16	48.5
	Strongly agree	10	30.3

Strategies to Enhance Travel Medicine Practice

**Figure 1:**
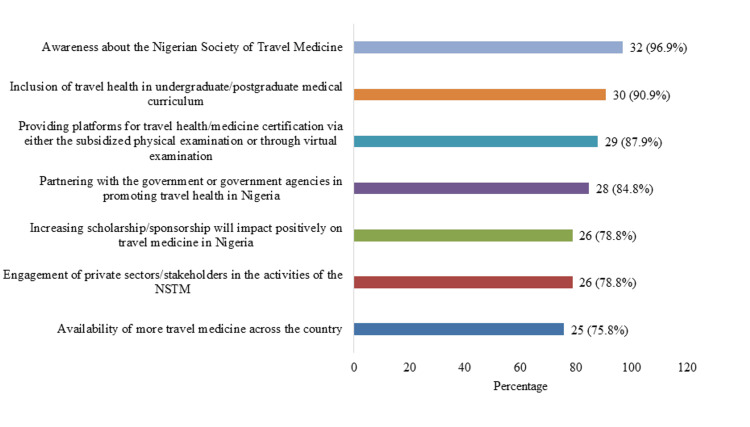
Things to do to improve/enhance the practice of travel medicine in Nigeria NSTM: Nigerian Society of Travel Medicine

Figure 1 illustrates respondents’ views on measures to improve travel medicine practice in Nigeria. Nearly all respondents (32, 97.0%) reported that increased awareness of the NSTM would enhance practice. Most (30, 90.9%) also identified the inclusion of travel health in undergraduate and postgraduate medical curricula, and 29 (87.9%) highlighted the provision of accessible certification platforms, either through subsidized in-person examinations or virtual options, as key strategies for strengthening travel medicine practice in the country.

## Discussion

The demographic profile of respondents contrasts with findings by Sharahili et al., where females predominated, and fewer respondents held consultant-level qualifications [12]. Most respondents practiced in south-western Nigeria, particularly Lagos, possibly due to proximity to the country’s busiest international airport [13].

The predominance of medical doctors among respondents aligns with findings by Kogelman et al., who reported that most pre-travel care providers were physicians, many specializing in family medicine [14]. Streit et al. similarly found that a large proportion of infectious disease specialists provided travel-related care [15].

Despite extensive practice experience, over two-thirds of respondents lacked formal certification in travel medicine. Similar findings have been reported in Australia and New Zealand [6]. Additionally, travel medicine thrives more in developed nations partly due to mandatory vaccination and prophylaxis requirements for travelers to tropical regions, including sub-Saharan Africa [16].

Certification costs and licensing challenges were major barriers identified. In terms of practice challenges, poor recognition and patronage were prominent. However, this is not unique to Nigeria, as Talbot et al. reported that a substantial proportion of travelers from developed regions do not seek pre-travel care [17].

Vaccine procurement, supply, storage, and cost were recurring challenges identified by the respondents. These findings are consistent with reports that vaccine costs limit the provision of pre-travel care [18].

Respondents were optimistic about the post-COVID era and the role of information technology, including telemedicine and electronic immunization data systems, which were successfully implemented during the COVID-19 response [7-8].

Limitation of the study

The results may not accurately reflect the views of the entire population of travel medicine practitioners in Nigeria due to the low response rate of the study, as well as the small sample size and geographical clustering of the respondents. There may also be a self-support bias whereby the challenges identified are erroneously blamed on external or situational factors.

Recommendation

More channels and opportunities should be provided for capacity building and certification of healthcare workers with an interest in travel medicine to ameliorate these challenges.

With the collaboration of the government and concerned non-governmental organizations (NGOs), proper attention and resources should be given to vaccine supply chain management.

A national policy recognition and regulatory framework to formally recognize travel medicine as a subspecialty area within Nigeria’s healthcare system is also recommended.

Government collaboration with professional bodies and NGOs should provide partial or full subsidies for internationally recognized certifications in travel medicine. Additionally, competitive grants or scholarships for physicians and nurses pursuing travel medicine training would also help.

The National Postgraduate Medical College of Nigeria and the West African College of Physicians should incorporate travel medicine modules into family medicine and community medicine residency curricula.

The government, in collaboration with immigration authorities, aviation agencies, travel agencies, and medical organizations, can also help with mass awareness and education to drive utilization of travel medicine services.

## Conclusions

This study evaluates the challenges and prospects of travel medicine practice in Nigeria. The newness of the specialty in Nigeria and poor public recognition were the most significant barriers identified. Future growth depends on increased awareness, professional acceptance, and strengthened service delivery models.

## Appendices

Appendix A

Questionnaire

A.    Socio-demographic characteristics

1.      Age at last birthday: _______ (years)

2.      Gender: Female _____, Male ______

3.      Religion: _______________

4.      Tribe: _____________

5.      Place of practice (City, State e.g Ikeja, Lagos) ________

6.      Highest Qualification: _________

B.     Professional Information

7.      What cadre of healthcare worker are you? Medical Doctor ___, Nurse___, Pharmacist ____, others ______

8.      What is your primary field of qualification (e.g Family Medicine, Public health physician, public health nurse, etc) ______________________

9.      How long have you been practicing Travel Health? _____ (years)

C.    Challenges of Travel Health

To what degree do you consider any of the following as a challenge in setting up a Travel Health Clinic?

**Table 7:** Questionnaire: Sections C, D, and E

		Strongly agree	Agree	Neutral	Disagree	Strongly disagree
10	Locating appropriate clinic site					
							11	Capital to start up the clinic					
12	Need for physical structure												
							13	Need to become a member of travel medicine society in my country					
14	Cost of writing the Travel Medicine Qualification Exams												
15	Passing the travel Medicine Qualification (licensing) Exams					
D	Challenges with the practice of Travel Medicine					
16	Travel Health is still new/green to most people in my community/area of practice					
17	I find it challenging or exhaustive providing repeated travelling advise to travellers					
18	The patronage of Traveller is poor and discouraging to fully engage in travel					
	medicine practice					
19	I believe many have not yet recognized travel medicine as a useful clinic					
20	I feel I cannot yet practice travel medicine alone without general medicine					
21	My city of residence is not ripe for travel medicine clinic yet					
E	The services of a travel medicine clinic					
22	Cost is an issue to potential traveller					
23	Our travel medicine clinics can adequately meet the need of every client					
24	We are able to cater for the disabled clients adequately					
25	Vaccine procurement is never an issue					
26	Vaccine supply chain is a serious issue					
27	Vaccine storage is never an issue					

Section F: Prospects of travel medicine practice in Nigeria

To what degree do you agree or disagree with the following concerning the prospect of travel medicine in Nigeria?

**Table 8:** Questionnaire: Section F

F		Strongly agree	Agree	Neutral	Disagree	Strongly disagree
28	The use of information technology will help the growth of travel health in Nigeria					
29	With the aid of information technology, the burden of initial start-up of travel medicine clinic will significantly reduce					
30	As was done for COVID-19 vaccination, an Electronic Management of Immunization Data (EMID) can also help with travellers vaccination monitoring and thus make the tracking easy for a travel medicine clinic.					
31	Development of software application for travel medicine practitioners will help to enhance their work rather than take away from it?					

32.    The following will help to improve/enhance the practice of travel medicine in Nigeria

a.       Awareness about the Nigerian Society of Travel Medicine (NSTM)

b.      Availability of more travel medicine across the country

c.       Inclusion of travel health in undergraduate/postgraduate medical curriculum

d.      Partnering with the government or government agencies in promoting travel health in Nigeria

e.       Engagement of private sectors/stakeholders in the activities of the NSTM

f.       Providing platforms for travel health/medicine certification via either the subsidized physical examination or through virtual examination

g.      Increasing scholarship/sponsorship will have a positive impact on travel medicine in Nigeria

33. Will the post-COVID era have a positive impact on travel health? Yes ___, No____.
